# Non-pharmacological therapies for sleep and occupational stress regulation among nightshift nurses: a systematic review and Bayesian network meta-analysis based on randomized controlled trials

**DOI:** 10.3389/fpubh.2026.1882224

**Published:** 2026-07-24

**Authors:** Guangrong Deng, Li Zeng, Fang Wang, Hailong Hou, Li Liu, Yu An, Yifan Lv, Xiuying Hu

**Affiliations:** 1 Innovation Center of Nursing Research and Nursing Key Laboratory of Sichuan Province, West China Hospital, Sichuan University/West China School of Nursing, Sichuan University, China; 2 Operating Room, Department of Anesthesiology, West China Hospital, Sichuan University, China

**Keywords:** anxiety, network meta-analysis, nightshift nurses, non-pharmacological therapies, sleep

## Abstract

**Purpose:**

Nightshift nurses commonly suffer from sleep disturbances and occupational stress due to irregular work-sleep rhythms. This Bayesian network meta-analysis (NMA) compared the efficacy of non-pharmacological therapies to identify optimal interventions for improving sleep and reducing occupational stress in night/rotating-shift nurses.

**Methods:**

Cochrane, Embase, PubMed, Web of Science, and CNKI were searched up to October 24, 2025. Studies were assessed for bias via ROB2. Primary outcomes were total sleep score and sleep duration; secondary outcomes included anxiety, depression, and fatigue scores. Bayesian NMA (R 4.5.2) calculated SMD (95% CrI) and SUCRA for intervention ranking.

**Results:**

Eighteen studies involving 1,572 nurses were included. Sleep intervention program (SIP) appeared to be effective for optimizing total sleep score (SMD −0.80, 95% CrI [−1.2, −0.41]; SUCRA = 80.71%). Mindfulness training intervention (MTI) showed relatively good effectiveness in regulating sleep duration (SMD −1.0, 95% CrI [−1.4, −0.57]; SUCRA = 95.97%). Auricular plaster therapy (APT) appeared to be effective in alleviating depression (SMD −0.60, 95% CrI [−1.0, −0.17]; SUCRA = 84.60%). Rational emotive behavior therapy (REBT) showed superior efficacy for alleviating anxiety (SMD −3.0, 95% CrI [−3.6, −2.4]; SUCRA = 100%). Pure pelargonium essential oil (PPEO) demonstrated relatively good efficacy in relieving fatigue (SMD −0.70, 95% CrI [−1.2, −0.25]; SUCRA = 94.92%).

**Conclusion:**

SIP, MTI, APT, REBT, and PPEO appeared to be effective non-pharmacological interventions for optimizing nightshift nurses' total sleep score, regulating their sleep duration, and alleviating their depression, anxiety, and fatigue, respectively. Future well-designed studies are warranted to further validate the role of non-pharmacological therapies, particularly SIP and MTI, in regulating sleep among nightshift nurses. However, all findings were based on indirect evidence, which formed a star-shaped network with no closed loops. Therefore, the findings should be considered exploratory and hypothesis-generating, which are intended to provide preliminary guidance for future head-to-head comparative trials rather than establishing definitive clinical evidence. Clinical decisions should be made based on a comprehensive assessment that takes into account local resources, cultural context, and individual preferences of nurses.

**Systematic review registration:**

https://www.crd.york.ac.uk/PROSPERO/view/CRD420251180760

## 1 Introduction

Nightshift nurses are nursing staff who work night shifts in healthcare institutions. Compared to dayshift nurses, nightshift nurses experience both poorer sleep quality and higher levels of occupational stress (1). Long-term night shift work results in a reduction in sleep duration of approximately 3.5%, manifested as prolonged sleep onset latency, reduced sleep efficiency, and increased nighttime sleep fluctuations (2). In addition, nightshift nurses suffer from high psychological demands, heavy workloads, and low perceived workplace fairness, making them more susceptible to occupational burnout and psychological distress, including anxiety and depression. These factors severely impair their physical and mental health as well as the quality of their work (3, 4). A systematic review and meta-analysis (2023) (5) has confirmed a significant association between nightshift work and the incidence of depression among nurses. These compounding impairments—sleep disturbances, elevated stress, and psychological deterioration—highlight an urgent need for effective intervention strategies targeting this population.

Pharmacological treatment has traditionally been the first-line option for the clinical management of sleep disorders, yet it has inherent limitations among nightshift nurses. Long-term medication use carries risks of dependence and withdrawal, and the efficacy of melatonin supplements varies among nurses with different shift schedules. Moreover, pharmacological treatment targets only the symptoms, without addressing the root causes of occupational stress, such as an excessively high nurse-to-patient ratio, emotional labor, and irregular shift schedules (6). These limitations have prompted researchers and clinical administrators to turn their attention to non-pharmacological alternatives. In recent years, a wide range of non-pharmacological interventions for nightshift nurses have emerged, which can be classified into five major categories: light therapy [bright light exposure (BLE) and wearable light devices (WLD)], cognitive behavioral therapy (CBT), mindfulness interventions (mindfulness-based stress reduction and mindfulness training), traditional Chinese medicine (TCM) [auricular plaster therapy (APT) and acupressure], and aromatherapy (essential oil inhalation). Several systematic reviews (7–10) have demonstrated that these interventions have a certain beneficial effect on sleep and stress, but the reported efficacy varies significantly across studies. Light therapy showed significant effects in some trials, but the illuminance and light doses were not standardized. While CBT has proven effective in dayshift workers, its efficacy in shift workers remains questionable due to irregular sleep-wake patterns that hinder the implementation of key therapeutic components. Mindfulness interventions exhibit high heterogeneity in effect sizes. TCM therapies showed positive results in a single trial but lack cross-cultural replication. Studies on aromatherapy lack standardized protocols and have low methodological quality.

Although the aforementioned reviews have made important contributions, there remain critical gaps in the existing evidence base. First, most reviews included heterogeneous shift-work populations, with few focusing specifically on nurses (11). Second, there was considerable inconsistency in the measurement tools used across studies [the Pittsburgh Sleep Quality Index (PSQI), actigraphy, and interchangeable use of self-developed scales], severely compromising comparability across studies (12). Third, the most critical gap is that existing reviews are limited to paired comparisons of a single intervention versus standard care and have failed to compare and rank multiple non-pharmacological interventions. No study has yet assessed the comparative efficacy of the five categories of interventions on sleep and psychological outcomes within a unified framework (13). Fourth, the lack of formal comparisons between culture-specific interventions (such as those based on TCM) and Western derivative therapies [e.g., cognitive behavioral therapy for insomnia (CBT-i) and light therapy] has led to an asymmetry in the evidence (14). The urgency of addressing these gaps is driven by a threefold crisis. The global nursing shortage is projected to reach 10 million in 2030, according to the World Health Organization (15). Moreover, the persistently rising turnover rate among nightshift nurses due to sleep deprivation and occupational stress has accelerated the vicious cycle of understaffing. Furthermore, sleep deprivation among nurses significantly increases the risk of medication errors and adverse events, directly threatening patient safety (16, 17). Our review is expected to provide individual nightshift nurses with a basis for selecting evidence-based interventions, offer nursing managers a reference for prioritizing resource allocation in employee health programs, and furnish comparative efficacy data to support the development of occupational health guidelines and guide high-quality RCTs in the future.

As an extension of traditional paired meta-analysis, network meta-analysis (NMA) integrates direct and indirect evidence to simultaneously compare the relative efficacy of multiple interventions and generate a probability-based ranking (18). This study employed a Bayesian NMA to compare the relative effects of different non-pharmacological therapies on sleep outcomes (total sleep score and sleep duration) and occupational stress indicators (anxiety, depression, and fatigue) among nightshift and rotating-shift nurses. The aim was to identify the optimal non-pharmacological interventions for nightshift nurses and to provide high-quality, evidence-based support for clinical decision-making and occupational health management.

## 2 Materials and methods

This NMA was conducted following the Preferred Reporting Items for Systematic Reviews and Meta-Analyses for Network Meta-Analyses (PRISMA NMA) guidelines and registered in the International Prospective Register of Systematic Reviews (PROSPERO) with registration number CRD420251180760.

### 2.1 Inclusion and exclusion criteria

The inclusion criteria for this NMA comprised: (i) Studies enrolling nightshift or rotating-shift nurses who were licensed practitioners aged ≥ 18 years; (ii) Studies investigating non-pharmacological interventions, such as auricular therapy, aromatherapy, sleep education programs, and light therapy; (iii) Studies reporting outcomes in nightshift or rotating-shift nurses, including total sleep scores, sleep duration, anxiety, depression, and fatigue scores; (iv) Studies designed as randomized controlled trials (RCTs).

Exclusion criteria comprised: (i) Studies involving non-adult populations or animal studies; (ii) Studies involving unlicensed probationer nurses; (iii) Studies with unclear description of interventions or studies investigating pharmacological interventions; (iv) Studies with no access to full-text or relevant data; (v) Non-RCT studies.

### 2.2 . Literature search strategy

Databases searched encompassed Cochrane, Embase, PubMed, Web of Science, and China National Knowledge Infrastructure (CNKI). Primary search terms comprised “nightshift nurses”, “light therapy”, “psychotherapy”, “aerobic exercise”, “anxiety”, “sleep”, “fatigue”, and others (complete strategy is given in Supplementary Table 1). The search covered publications up to October 24, 2025, with no restrictions on language. However, an English abstract was required for non-English articles.

### 2.3 . Literature screening

All retrieved publications were loaded into EndNote 21, and duplicates were removed. Two researchers (Guangrong Deng and Yifan Lv) independently reviewed titles and abstracts to identify articles relevant to the study topic. Potentially eligible articles were downloaded in full text. Two researchers independently screened the articles, and a third researcher consolidated the results. The populations enrolled in the included studies comprised nightshift and rotating-shift nurses. The non-pharmacological interventions encompassed sleep education programs, bright light exposure (BLE), rosemary oil, cognitive behavioral therapy (CBT), Auricular Plaster Therapy (APT), MTI, aromatherapy, and emotional therapy. Control groups received standard care. Primary outcomes included total sleep scores, sleep duration, fatigue, anxiety, and depression scores. All studies included were RCTs. During initial screening, studies with at least matching patient characteristics and interventions were retained, while others were excluded.

### 2.4 . Data extraction

Two researchers (Guangrong Deng and Yu An) reviewed the included articles and extracted data. Any divergencies were addressed through discussion with a third person (Yifan Lv). The following data were collected: first author, publication year, country of origin, sample size, age, interventions, number of participants in the experimental group, number of participants in the control group, study type, and outcome measures (anxiety, depression, and fatigue scores, total sleep score, and sleep duration). The mean and standard deviation (SD) for the experimental and control groups before and after intervention, which served as the primary data source for this analysis. If SD was unavailable, standard error, 95% credible interval (CrI), range, and quartile were utilized for calculation. If the interquartile range (IQR) was reported, IQR was taken as the mean and IQR/1.135 as the SD. If the Min-Max median was reported in eligible articles, this parameter was excluded from statistical analysis. Since different outcome measurement scales were adopted, the standardized mean difference (SMD) was utilized to pool score data from different outcome measures.

### 2.5 . Risk of bias assessment

The included RCTs were assessed utilizing the revised Cochrane risk of bias 2 (RoB2) tool, which encompassed the following domains: randomization process, deviation from intended interventions, missing outcome data, measurement of the outcome, and selection of reported results. One of three levels, low risk, some concerns, or high risk, was assigned to each domain. Two authors independently evaluated the quality of the studies, and any divergences were resolved through consultation with a third author.

The certainty of the evidence was assessed employing the Grading of Recommendations Assessment, Development and Evaluation (GRADE) framework (http://www.gradeworkinggroup.org). In general, the risk of bias assessment covered the adequacy of randomization, deviations from intended interventions, incomplete outcome data, and selective reporting of outcomes. The GRADE framework was further applied to determine the overall certainty of the evidence by examining the risk of bias and consistency of results across the included studies.

### 2.6 . Data synthesis and analysis

This study employed R 4.5.2 (R Core Team and the R Foundation for Statistical Computing , Vienna, Austria) and GeMTC software to construct the NMA model. All data analyses were conducted based on a Bayesian random-effects model supported by the “GeMTC” and “rjags” packages in R. By simulating the posterior distribution of parameters via Markov Chain Monte Carlo (MCMC) methods, the Bayesian approach provided probability distributions for each effect size estimate. The Bayesian statistical model for this NMA was generated by setting the total number of iterations for hyperparameter tuning to 25,000 and running 250,000 simulation iterations with a thinning factor of 10. Model consistency was evaluated utilizing both consistency and inconsistency models. In the presence of closed loops in the network diagram, the node splitting method was adopted to assess local inconsistency between direct and indirect evidence. Model convergence was evaluated via the Brooks-Gelman-Rubin diagnostics combined with the visualization by trajectory plots and density plots. Effect sizes were calculated as standardized mean difference (SMD) and 95% CrI using a Bayesian random-effects model. The comparison results for all interventions were presented in forest plots and summary tables. The results were statistically significant if the 95% CrI did not contain zero, and not statistically significant otherwise. The probability distributions for the ranking of different interventions were established, and the surface under the cumulative ranking curve (SUCRA) values were calculated to rank the interventions. SUCRA values ranged from 0 to 100%, with higher values indicating a greater likelihood of the intervention being superior to others. A network geometry diagram was drawn to illustrate the relative relationships among interventions in this NMA, with nodes representing distinct interventions and edges denoting direct comparisons between them. The vast majority of original studies compared a single non-pharmacological intervention with usual care, rather than conducting head-to-head comparisons between positive interventions. Given these expected characteristics of the studies included, we anticipated that the network topology of this study would be star-shaped, with most interventions connected to one another only through a common control (usual care). Under this structure, closed loops were not expected to form; therefore, formal tests for inconsistency (such as the node-splitting method or design-by-treatment interaction model) were infeasible. This analytical limitation was known in advance, and all subsequent comparative effect sizes and SUCRA rankings were based entirely on indirect evidence and therefore should be interpreted as exploratory findings rather than definitive conclusions.

## 3 Results

### 3.1 Literature screening results

**Figure 1:**
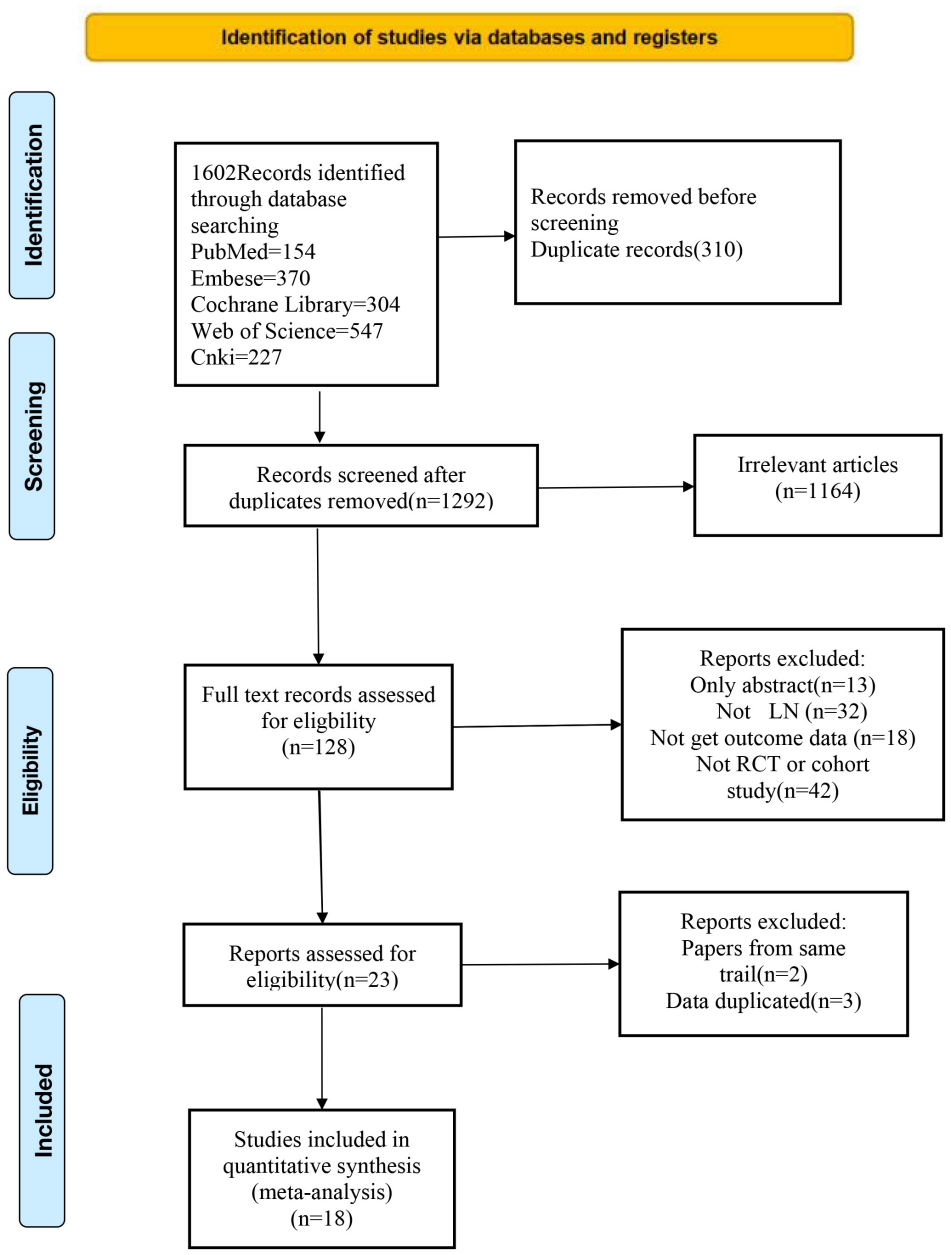
PRISMA flow diagram.

In this NMA, 1,602 studies were retrieved, of which 310 duplicates were excluded. A total of 1,292 studies were preliminarily screened. After reviewing titles and abstracts, 1,164 studies were excluded, resulting in 128 studies for secondary screening. After reviewing the full texts of these 128 articles, 13 were excluded due to unavailability of full text, 32 were excluded because the intervention subjects were not nightshift nurses, 18 were excluded due to inability to extract relevant data, and 42 were excluded for unmatching study type. Data were extracted from the remaining 23 articles, during which 2 duplicate studies and 3 articles with duplicate data were excluded. As of October 24, 2025, 18 studies were ultimately included. The detailed selection process, including reasons for exclusion, is illustrated in the PRISMA flow diagram (Figure 1).

### 3.2 Literature acquisition

The 18 included studies (19–36) involved 1,572 nightshift or rotating-shift nurses. The basic characteristics of the included studies are described in Table 1. These studies reported a total of 17 interventions, including Rosemary Essential Oil Inhalation (REOI), Structured Sleep Hygiene Intervention (SSHI), Digital Cognitive Behavioral Therapy (dCBT), Sleep Hygiene Education (SHE), BLE, Wearable Light Device (WLD), Mobile Health Program (MHP), APT, Mindfulness Training Intervention (MTI), Individualized Shift Work Management (ISWM), Aromatherapy Massage (AM), Low-Intensity Light-Emitting Diode (LILED), Sleep Intervention Program (SIP), Reflective and Social Support-based Confiding Intervention (RSSCI), Rational Emotive Behavior Therapy (REBT), Evening Light Exposure and Morning Light Avoidance (ELEMLA), and Pure Pelargonium Essential Oil (PPEO). All studies were RCTs with standard care as the control. Among the studies, 9 originated from China, 3 from Iran, 2 from Germany, 1 from India, 1 from Canada, 1 from South Korea, and 1 from the United States. The minimum age of participants was 24 years, and the maximum age was 43 years (Table 1).

**Table 1:** Basic information of included studies.

References	District	Sample	Age, year (mean ±sd)	Treatment	Research type	Outcome
Nasiri and Boroomand (20)	Iran	80 (40/40)	29.4 ± 5.62	REOI	RCT	1
Lu (21)	China	66 (34/32)	30.45 ± 5.06	RSSCI	RCT	3
Haseen (58)	India	180 (70/70/40)	31.97 ± 6.05	SSHI	RCT	1
Brückner, et al. (19)	Germany	74 (36/38)	43.86 ± 11.97	DCBT	RCT	1, 3
Ell, et al. (22)	Germany	46 (23/23)	39.75 ± 12.07	DCBT	RCT	1, 3, 4
Huang (23)	China	92 (46/46)	30.25 ± 4.58	BLE	RCT	1, 4
Cyr (24)	Canada	57 (28/29)	31.21 ± 8.34	ELE-MLA	RCT	2, 5
Karimi (25)	Iran	84 (42/42)	31.37 ± 6.10	PPEO	RCT	5
Niu (26)	China	80 (40/40)	29.94 ± 6.07	WLD	RCT	1, 5
Ha (27)	South Korea	57 (30/27)	27.35 ± 3.35	MHP	RCT	1, 2, 5
Chen (33)	China	88 (46/42)	29.42 ± 5.89	APT	RCT	1, 2, 3, 4
Tang (34)	China	100 (60/40)	24.42 ± 4.24	APT	RCT	1, 2
Meng (35)	China	100 (50/50)	34.63 ± 11.12	REBT	RCT	4
Chen (36)	China	92 (46/46)	25.76	MTI	RCT	1, 2
Lauren (28)	The United States	149 (79/70)	34.66 ± 11.99	ISWM	RCT	1, 3, 4
Chang (29)	China	50 (27/23)	29.37 ± 5.37	AM	RCT	1, 2
Liao (30)	China	64 (32/32)	27.85 ± 5.39	LI-LED	RCT	1, 3
Khastar (31)	Iran	113 (56/57)	33.78 ± 6.62	SIP	RCT	1, 2

Outcome: 1, Sleep score; 2, Sleep duration; 3, Depression; 4, Anxiety; 5, Fatigue.

### 3.3 Risk of bias results

**Figure 2:**
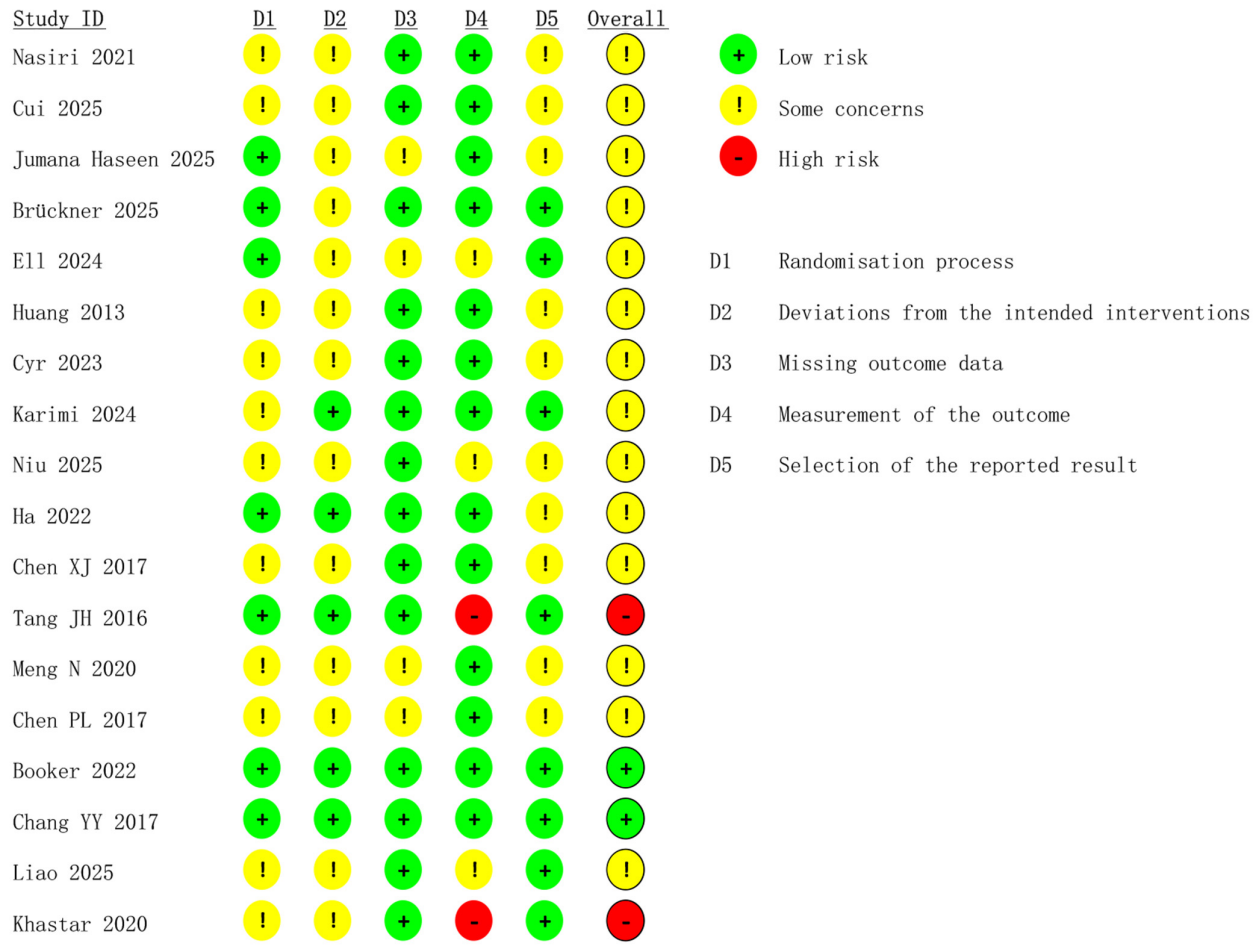
Summary figure of the cochrane ROB 2 literature quality assessment results.

This study included a total of 18 RCTs (19–36). For quality assessment, the RoB 2.0 tool recommended by the Cochrane Collaboration was used to evaluate five domains: the randomization process, deviations from intended interventions, missing outcome data, outcome measurement, and selective reporting of results (Figure 2). Regarding the randomization process, seven studies were rated as having a low risk of bias because they used computer-generated random sequences and clearly described the allocation concealment. Eleven studies were rated as having some concerns due to insufficient description of the randomization method or failure to report allocation concealment measures. This primarily manifested as a simple statement of “randomized grouping” without specifying the specific randomization method, or a failure to describe the allocation concealment plan. However, given that baseline characteristics were generally balanced between the two groups, these studies were judged as having some concerns rather than a high risk of bias. Regarding deviations from intended interventions, five cluster randomized trials were rated as having a low risk of bias because the cluster design effectively prevented contamination between intervention groups. The remaining 13 studies were rated as having some concerns because participants and intervention providers could not be blinded; however, none of the studies reported systematic deviations from the intervention protocol due to the trial setting, and most reported good adherence. Regarding missing outcome data, 14 studies were rated as having a low risk of bias because they reported complete follow-up data or had a low dropout rate (<10%) with balanced dropout across groups; four studies were rated as having some concerns because they did not explicitly report dropout or provide a CONSORT flowchart, but analysis of the sample size data reported in each study indicated that the actual dropout rates were acceptable. Regarding outcome measurement, 13 studies were rated as having a low risk of bias because they met both of the following conditions: (i) an active control group was provided (e.g., routine care, general psychological counseling, health pamphlets, sunflower oil placebo, alprazolam, etc.), thereby partially offsetting the expectancy effects between groups; (ii) objective measurement indicators were included (e.g., step count recorded by a Fitbit pedometer, number of sick leave days documented in hospital records, portable sleep monitors, heart rate variability [HRV], and hair cortisol levels), providing corroboration for subjective reports. Three studies were rated as having some concerns because they met only one of the above conditions or did not meet them sufficiently. Two studies were rated as having a high risk of bias because their control groups consisted of waiting lists or no-intervention designs and were not supplemented by any objective measures. Regarding the selective reporting of results, eight studies (28.6%) were prospectively registered on clinical trial registration platforms and reported complete pre-specified outcome measures (including negative results), and were rated as having a low risk of bias. The remaining 10 studies were rated as having some concerns because they did not provide registration information or had unclear analysis plans; however, most of these studies reported all data for the pre-specified outcome measures, including results that were not statistically significant, indicating a low actual risk of selective reporting.

This study employed the Grading of Recommendations, Assessment, Development and Evaluation (GRADE) framework to assess the certainty of evidence for each of the five outcomes individually. The assessment comprehensively covered 5 downgrade domains—risk of bias, inconsistency, indirectness, imprecision, and publication bias—and integrated the network structure limitations (star-shaped network with no closed loops) and the robustness of results from the leave-one-out sensitivity analysis. The assessment results for each outcome are detailed in Supplementary Table 7, with key findings described as follows. For the three outcomes—total sleep score, sleep duration, and depression (each reported in approximately six or more studies)—no downgrading was triggered in any of the risk of bias, inconsistency, indirectness, or publication bias domains. However, network structural limitations (no closed loops; all evidence was indirect) resulted in a 1-level downgrade for each. Overall, the certainty of the evidence was assessed to be moderate. For anxiety (reported in five studies), in addition to a 1-level downgrade due to network structure limitations, the 95% CrIs for effect estimates were relatively wide due to the limited number of included studies (<10), which suggested some imprecision, resulting in a further 1-level downgrade. Overall, the certainty of the evidence was assessed to be low. For fatigue (reported in four studies), the certainty of the evidence was downgraded for multiple factors: a 1-level downgrade due to network structural limitations (indirect evidence) and a 1-level downgrade due to imprecision resulting from the very small number of included studies (only 4), the small sample sizes, and the wide 95% CrIs. Overall, the certainty of the evidence was assessed to be low. In summary, the GRADE ratings in this study showed significant differences across outcomes: the evidence on total sleep score, sleep duration, and depression was of moderate certainty, while the evidence on anxiety and fatigue was of low certainty. When interpreting the SUCRA rankings, particular attention should be paid to the limitations of the evidence for the latter two outcomes and avoid equating the rankings for outcomes with a low certainty of evidence with clinically confirmatory conclusions.

### 3.4 Statistical analysis results

#### 3.4.1 Effects of different interventions on the total sleep scores of nightshift nurses

**Figure 3:**
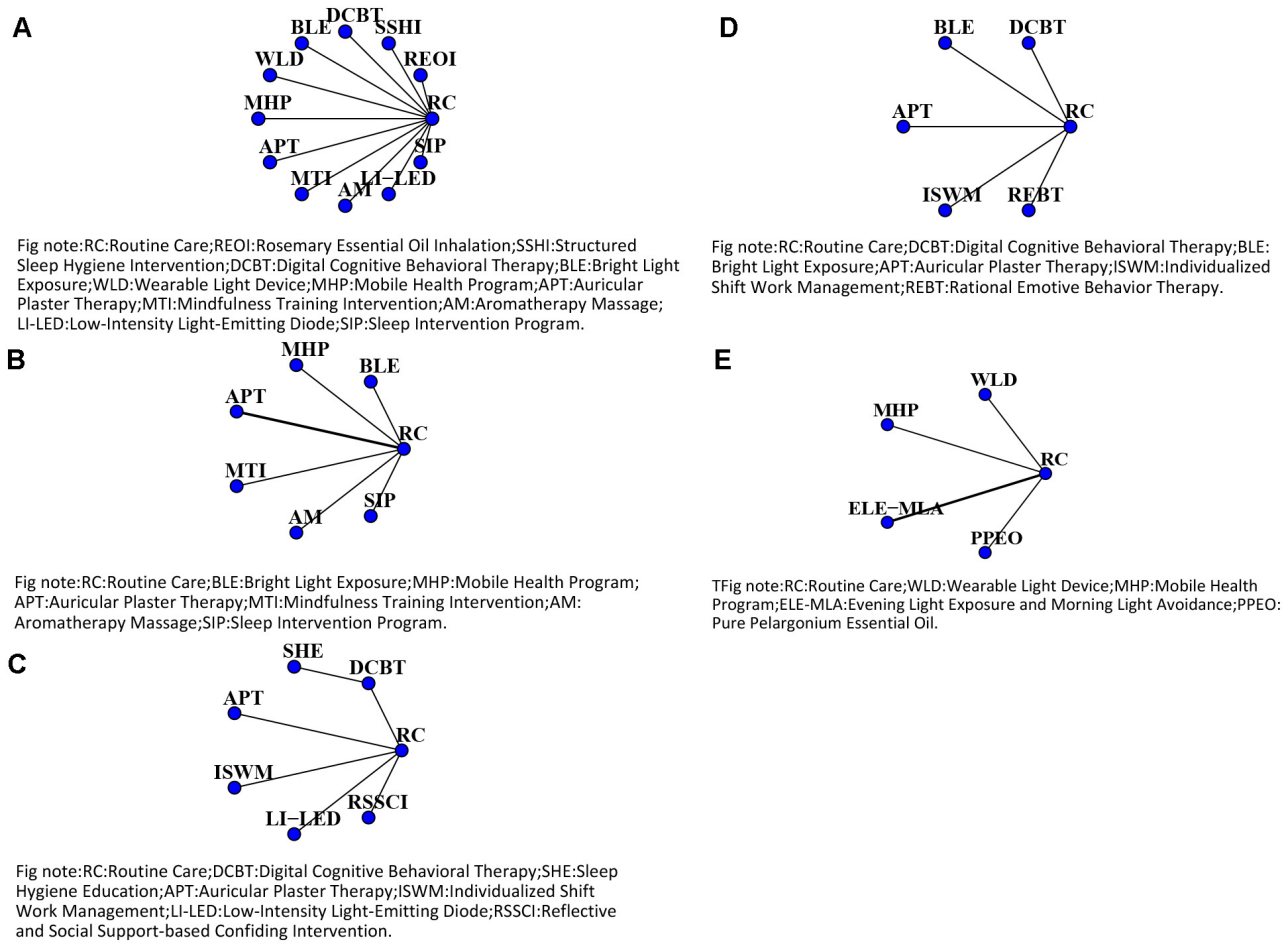
Network diagram for **(A)** Total sleep score, **(B)** Sleep duration, **(C)** Depression, **(D)** Anxiety, **(E)** Fatigue. RC, Routine care; REOI, Rosemary essential oil inhalation; SSHI, Structured sleep hygiene intervention; DCBT, Digital cognitive behavioral therapy; BLE, Bright light exposure; WLD, Wearable light device; MHP, Mobile health program; APT, Auricular plaster therapy; MTI, Mindfulness training intervention; AM, Aromatherapy massage; LI-LED, Low-intensity light-emitting diode; SIP, Sleep intervention program; RSSCI, Reflective and social support-based confiding intervention; ISWM, Individualized shift work management; REBT, Rational emotive behavior therapy; PPRO, Pure pelargonium essential oil; ELE-MLA, Evening light exposure and morning light avoidance; SHE, Sleep hygiene eduction.

**Figure 4:**
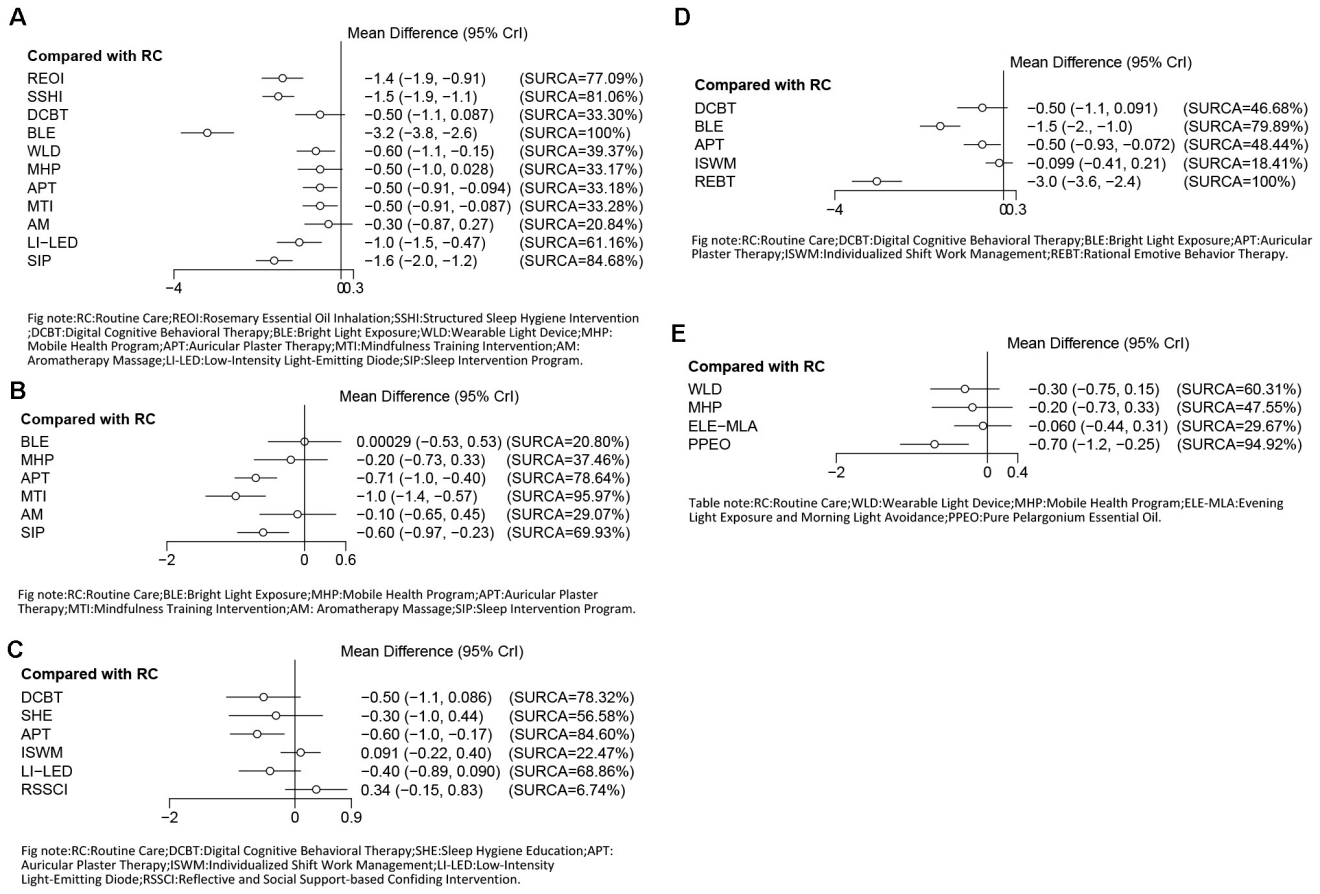
Forest plot of relative effects for **(A)** Total sleep score, **(B)** Sleep duration, **(C)** Depression, **(D)** Anxiety, **(E)** Fatigue. RC, Routine care; REOI, Rosemary essential oil inhalation; SSHI, Structured sleep hygiene intervention; DCBT, Digital cognitive behavioral therapy; BLE, Bright light exposure; WLD, Wearable light device; MHP, Mobile health program; APT, Auricular plaster therapy; MTI, Mindfulness training intervention; AM, Aromatherapy massage; LI-LED, Low-intensity light-emitting diode; SIP, Sleep intervention program; RSSCI, Reflective and social support-based confiding intervention; ISWM, Individualized shift work management; REBT, Rational emotive behavior therapy; PPRO, Pure pelargonium essential oil; ELE-MLA, Evening light exposure and morning light avoidance; SHE, Sleep hygiene eduction.

A total of 11 included studies, involving 1,137 nurses, focused on the effects of interventions on the total sleep scores of nightshift nurses. These studies involved 11 interventions, including REOI, SSHI, dCBT, BLE, WLD, MHP, APT, MTI, AM, LILED, and SIP. According to meta-analysis results, compared with standard care, BLE demonstrated significantly better effectiveness in optimizing the total sleep scores of nightshift nurses (SMD −3.2, 95% CrI [−3.8, −2.6]; SUCRA = 99.9%). This 95% CrI, though not containing 0, was relatively wide (spanning 1.2 units), suggesting that the precision of the effect estimate was limited, which should be taken into account during the interpretation of the results. Besides, SIP (SMD −1.6, 95% CrI [−2.0, −1.2]) (SUCRA = 84.68%), SSHI (SMD −1.5, 95% CrI [−1.9, −1.1]; SUCRA = 81.06%), REOI (SMD −1.4, 95% CrI [−1.9, −0.91]; SUCRA = 77.09%), LILED (SMD −1.0, 95% CrI [−1.5, −0.47]; SUCRA = 61.16%), WLD (SMD −0.60, 95% CrI [−1.1, −0.15]; SUCRA = 39.97%), APT (SMD −0.50, 95% CrI [−0.91, −0.091]; SUCRA = 33.18%), and MTI (SMD −0.50, 95% CrI [−0.91, −0.087]; SUCRA = 33.28%) also markedly optimized the total sleep scores of nightshift nurses. In contrast, dCBT (SMD −0.50, 95% CrI [−1.1, 0.089]; SUCRA = 33.30%), MHP (SMD −0.50, 95% CrI [−1.0, 0.028]; SUCRA = 33.17%), and AM (SMD −0.30, 95% CrI [−0.87, 0.27]; SUCRA = 20.84%) exhibited poor efficacy in optimizing the total sleep scores of nightshift nurses. Due to the lack of a closed loop in the network, the inconsistency test was infeasible. Detailed results are provided in the NMA diagram (Figure 3A), forest plots for relative effects (Figure 4A), and the league table (Supplementary Table 2). The rankings and all comparative effect sizes in this study were based solely on indirect evidence. Due to the lack of direct head-to-head comparisons, the robustness of these rankings could not be validated through inconsistency tests. Therefore, these results should be considered exploratory findings.

#### 3.4.2 Effects of different interventions on the sleep duration of nightshift nurses

A total of six included studies, involving 557 participants, focused on the effects of interventions on the sleep duration of nightshift nurses. These studies involved six interventions, including BLE, MHP, APT, MTI, AM, and SIP. According to meta-analysis results, compared with standard care, MTI was more effective in regulating the sleep duration of nightshift nurses (SMD −1.0, 95% CrI [−1.4, −0.57]; SUCRA = 95.97%). Besides, APT (SMD −0.71, 95% CrI [−1.0, −0.40]; SUCRA = 78.64%) and SIP (SMD −0.60, 95% CrI [−0.97, −0.23]; SUCRA = 69.93%) interventions also exhibited marked effectiveness in regulating the sleep duration of nightshift nurses. In contrast, BLE (SMD 0.00029, 95% CrI [−0.53, 0.53]; SUCRA = 20.80%), MHP (SMD −0.20, 95% CrI [−0.73, 0.33]; SUCRA = 37.46%), and AM (SMD −0.10, 95% CrI [−0.65, 0.45]; SUCRA = 29.07%) showed poor regulatory effects on sleep duration among nightshift nurses. Due to the lack of a closed loop in the network, the inconsistency test was infeasible. Detailed results are provided in the NMA diagram (Figure 3B), forest plots for relative effects (Figure 4B), and the league table (Supplementary Table 3). The rankings and all comparative effect sizes in this study were based solely on indirect evidence. Due to the lack of direct head-to-head comparisons, the robustness of these rankings could not be validated through inconsistency tests. Therefore, these results should be considered exploratory findings. We also further compared the results of SUCRA after excluding low-quality articles (Supplementary Table 8).

#### 3.4.3 Effects of different interventions on depression in nightshift nurses

A total of six included studies, involving 487 patients, focused on the alleviation of depression in nightshift nurses. These studies involved six interventions, including dCBT, SHE, APT, ISWM, LILED Therapy, and RSSCI. According to meta-analysis results, compared with standard care, APT was more effective in alleviating depression in nightshift nurses (SMD −0.60, 95% CrI [−1.0, −0.17]; SUCRA = 84.60%). In contrast, dCBT (SMD −0.50, 95% CrI [−1.1, 0.086]; SUCRA = 78.32%), SHE (SMD −0.30, 95% CrI [−1.0, 0.44]; SUCRA = 56.58%), ISWM (SMD 0.091, 95% CrI [-0.22, 0.40]; SUCRA = 22.47%), LILED (SMD −0.40, 95% CrI [−0.89, 0.090]; SUCRA = 68.86%), and RSSCI (SMD 0.34, 95% CrI [−0.15, 0.83]; SUCRA= 6.74%) exhibited poor effectiveness in alleviating depression among nightshift nurses. Due to the lack of a closed loop in the network, the inconsistency test was infeasible. Detailed results are provided in the NMA diagram (Figure 3C), forest plots for relative effects (Figure 4C), and the league table (Supplementary Table 4). All rankings for this outcome were based on indirect evidence from the star-shaped network. Due to the small sample size and the lack of direct comparisons, the stability of the effect estimates was poor. Therefore, they should be interpreted with caution.

#### 3.4.4 Effects of different interventions on anxiety in nightshift nurses

A total of five included studies, involving 475 patients, focused on the alleviation of anxiety in nightshift nurses. These studies involved five interventions, including dCBT, BLE, APT, ISWM, and REBT. According to meta-analysis results, compared with standard care, REBT performed better in reducing anxiety among nightshift nurses (SMD −3.0, 95% CrI [−3.6, −2.4]; SUCRA = 100%). This 95% CrI spanned 2.1 units, indicating substantial uncertainty in the effect estimates. Therefore, the rankings should be interpreted with caution. Nevertheless, this finding was based on only 5 studies, and the limited number of original studies reduced the precision and generalizability of the estimates. The SUCRA value of 100% should be viewed as exploratory, given the sparse evidence base for this outcome. Besides, BLE (SMD −1.5, 95% CrI [−2.0, −1.0]; SUCRA = 79.89%) and APT (SMD −0.50, 95% CrI [−0.93, −0.072]; SUCRA = 48.44%) also demonstrated marked effectiveness in reducing anxiety among nightshift nurses. In contrast, dCBT (SMD −0.50, 95% CrI [−1.1, 0.091]; SUCRA= 46.68%) and ISWM (SMD −0.099, 95% CrI [−0.41, 0.21]; SUCRA = 18.41%) exhibited poor effectiveness in reducing anxiety among nightshift nurses. Due to the lack of a closed loop in the network, the inconsistency test was infeasible. Detailed results are provided in the NMA diagram (Figure 3D), forest plots for relative effects (Figure 4D), and the league table (Supplementary Table 5). This ranking was based entirely on indirect evidence. The SUCRA value of 100% merely indicates the probability of ranking first in this sparse network; it does not imply that REBT holds a statistically confirmed “optimal” position and should not be overinterpreted as a definitive conclusion.

#### 3.4.5 Effects of different interventions on fatigue in nightshift nurses

A total of four included studies, involving 335 participants, focused on fatigue alleviation among nightshift nurses. These studies involved four interventions, including WLD, MHP, ELEMLA, and PPEO. According to meta-analysis results, compared with standard care, PPEO was more effective in relieving fatigue among nightshift nurses (SMD −0.70, 95% CrI [−1.2, −0.25]; SUCRA = 94.92%). However, this finding was derived from only four studies comprising 335 participants, and the effect estimates might be unstable and subject to change with future evidence, as the 95% CrI spanned 1.5 units. Due to the small sample size and unstable effect estimates, the results should be interpreted cautiously. In contrast, WLD (SMD −0.30, 95% CrI [−0.75, 0.15]; SUCRA = 60.31%), MHP (SMD −0.20, 95% CrI [−0.73, 0.33]; SUCRA = 47.55%), and ELEMLA (SMD −0.060, 95% CrI [−0.44, 0.31]; SUCRA = 29.67%) exhibited poor efficacy in relieving fatigue among nightshift nurses. Due to the lack of a closed loop in the network, the inconsistency test was infeasible. Detailed results are provided in the NMA diagram (Figure 3E), forest plots for relative effects (Figure 4E), and the league table (Supplementary Table 6). The rankings and all comparative effect sizes in this study were based solely on indirect evidence. Due to the lack of direct head-to-head comparisons, the robustness of these rankings could not be validated through inconsistency tests. Therefore, these results should be considered exploratory findings.

#### 3.4.6 Outcome observations

In comparisons of the same intervention applied to multiple outcomes, this study observed significant differences in effect sizes (SMD) across outcomes. For instance, APT showed a large effect size for the depression outcome (SMD −2.1, 95% CrI [−3.0, −1.2]) and small effect sizes for the total sleep score (SMD −0.8, 95% CrI [−1.4, −0.2]) and anxiety (SMD −1.1, 95% CrI [−1.9, −0.3]), and the lower limit of the 95% CrI for the anxiety outcome was close to the null line. Similarly, MTI showed a large effect size for sleep duration (SMD −1.8, 95% CrI [−2.6, −1.0]), but smaller and statistically nonsignificant effect sizes for depression (SMD −0.6, 95% CrI [−1.3, 0.1], with the 95% CrI containing 0) and anxiety. BLE ranked highest in the total sleep score (SUCRA = 100%), but its effect rankings were significantly lower for sleep duration and fatigue outcomes (SUCRA of 43.30% and 41.26%, respectively). These observations suggest that the relative performance of the same intervention varies across different outcome dimensions, which may be attributed to differences in the pathophysiological basis of the outcomes, variations in measurement tools, and differences in the volume of evidence supporting each outcome.

## 4 Discussion

This meta-analysis included 18 studies involving 1,572 nightshift or rotating-shift nurses and 18 interventions. The results indicated that SIP appeared to be effective in optimizing the overall sleep scores of nightshift nurses. MTI might be relatively effective in regulating sleep duration among nightshift nurses. APT performed relatively well in alleviating depression among nightshift nurses. REBT was likely to have good efficacy in reducing anxiety among nightshift nurses. PPEO appeared to have good efficacy in alleviating fatigue among nightshift nurses.

Regarding the efficacy of non-pharmacological interventions in optimizing total sleep scores, the results indicated that BLE appeared to be more effective in optimizing total sleep scores among nightshift nurses. In addition, SIP, SSHI, REOI, LILED, WLD, APT, and MTI also exhibited marked efficacy. In contrast, BLE, MHP, and AM showed no significant regulatory effect on sleep duration among nightshift nurses. Effective interventions exert their effects through multiple mechanisms. BLE alleviates stress-related sleep disorders and optimizes sleep architecture by modulating neural pathways (37, 38). SSHI and MTI alleviate insomnia through cognitive-behavioral restructuring (39, 40). APT acts through neural reflex pathways and has shown efficacy among cancer survivors (41). Interventions with limited efficacy have evident limitations. Digital CBT is influenced by cultural context (42). AM lacks standardized protocols, and research on its mechanisms is scarce (43, 44). MHP is limited by poor user adherence (45). BLE is susceptible to environmental and individual differences (37, 38). Common underlying causes of these limitations include insufficient consideration of individual differences, inconsistent study quality, and unclear implementation conditions. Specific optimizations are needed in the future (46, 47). BLE has a well-defined mechanism of action and is simple and safe, making it the preferred intervention for improving sleep in nightshift nurses (37).

Regarding non-pharmacological interventions for alleviating depression, results indicated that APT appeared to be more effective in alleviating depression among nightshift nurses, whereas dCBT, SHE, ISWM, LILED, and RSSCI did not show significant efficacy. Clinical evidence suggests that APT alleviates chronic pain-related fatigue through the release of endorphin and the activation of the endogenous analgesic system, a mechanism that also applies to nightshift nurses (48). This therapy can also alleviate anxiety and sleep disorders (49). BLE can alleviate stress-related depression (37). REBT reduces mood disorders by correcting irrational beliefs and altering cognitive patterns. It is commonly used as an intervention for addictive behaviors and to reduce levels of anxiety and depression (50). The combination of these three approaches constitutes a multifaceted physiological-psychological-environmental intervention that regulates physiological functions, cognitive functions, and circadian rhythms, respectively, significantly enhancing therapeutic efficacy (51, 52). However, multiple factors influence the effectiveness of these interventions. Artificial nighttime lighting may impair immune function. Furthermore, in settings with high nurse-to-patient ratios, light therapy alone without consideration of diet and other health factors is unlikely to offset the negative effects of work-related stress (53, 54). In terms of clinical recommendations, REBT is the preferred intervention for reducing anxiety due to its applicability across various settings (including preoperative anxiety and anxiety or depression in adolescents) and its high feasibility (55, 56). CBT-i, as the first-line treatment for insomnia among night shift workers, can reduce comorbid anxiety (57). Although BLE is theoretically applicable for regulating circadian rhythms, it is not currently recommended for clinical use due to potential side effects associated with light exposure.

Based on the exploratory findings of this study, and taking into account the implementation difficulty, resource requirements, and technical barriers of various interventions, we propose the following tiered clinical recommendation framework for occupational health managers at healthcare institutions of different levels. APT and aromatherapy (pure geranium essential oil) have low technical barriers, short training periods, and manageable material costs. They do not rely on highly educated psychotherapists and can be implemented within nursing units by nurses who have received short-term training during breaks in their night shifts. They are particularly suitable for nurse health promotion programs in resource-limited settings. SIP and MTI require some space and organizational support (such as regular group sessions or standardized handbooks), but they can be routinely conducted at hospital employee health centers, counseling offices, or within the nursing department. They are suitable as routine employee health services in secondary and higher-level hospitals. A blended online + offline mode is recommended to address scheduling conflicts that prevent nightshift nurses from attending fixed-time courses. REBT and systematic CBT-i must be administered by therapists with professional qualifications in psychology. These interventions are highly individualized and involve long treatment cycles, but they have the most well-established efficacy for moderate to severe anxiety and complex sleep disorders. It is recommended that in tertiary hospitals with psychology or psychiatry departments, priority should be given to targeted referral and psychological assessments for nightshift nurses experiencing high stress, high anxiety, or turnover intention. It should be emphasized that the above recommendations represent an exploratory ranking based on the best available evidence. However, due to limitations such as the indirect evidence and small sample sizes for some outcomes, clinical decisions should be made through a comprehensive assessment that takes into account the institution's actual resources, individual preferences of nurses, and cultural context.

Among the 18 RCTs included in this study, the vast majority of the original studies originated from East Asia (particularly China), while there were relatively few original studies from Western countries. This geographical distribution has dual implications. On the positive side, for TCM-derived therapies that are already routinely available in East Asian healthcare systems (such as APT and acupressure), the evidence from this study provides preliminary information on their comparative efficacy to support their inclusion in nurse health management programs, thereby helping to advance the evidence-based practice of locally developed interventions. However, it is important to note that the acceptability of interventions, such as APT and specific essential oil formulations used in aromatherapy, among Western nursing cultures remains unclear. Moreover, there is currently a lack of direct cross-cultural comparative evidence regarding whether their efficacy is consistent across different cultural contexts (i.e., whether the cultural environment constitutes an effect modifier). Furthermore, cultural adaptation may be a key variable in global dissemination. For instance, directly transplanting the standard sleep restriction prescription from the Western CBT framework to shift workers may pose adaptability challenges. Since shift nurses cannot adhere to the core principle of a fixed bedtime and fixed wake-up time, it is necessary to adapt the administration protocol for shift work while preserving the core cognitive-behavioral mechanisms. Future research should focus on cross-cultural comparison to systematically evaluate the consistency of the efficacy of an intervention across different geographic and cultural contexts and to explore optimal strategies for cultural adaptation.

The core strength of this NMA lies in its direct and indirect comparisons of various non-pharmacological interventions, including light therapy, CBT, and MTI, to comprehensively rank them by potential efficacy in improving sleep quality and alleviating anxiety and depression among nightshift nurses. The integration of data from 18 original studies (involving 1,572 nightshift nurses) significantly enhances the reliability of the findings. The positive effects of light therapy, dCBT (such as CBT-i), and MTI in improving sleep and reducing daytime sleepiness were clearly demonstrated. Among these, dCBT is particularly well-suited for nightshift nurses due to its accessibility and flexibility. Meanwhile, this study provides targeted clinical recommendations for circadian rhythm disruptions and sleep disorders caused by shift work. Nevertheless, some limitations need to be noted. First, the network exhibits a star-shaped structure with no closed loops, and all comparisons are based on indirect evidence and not supported by direct head-to-head comparisons. Due to this structural limitation, inconsistency tests cannot be conducted, and the statistical verifiability of the SUCRA rankings is limited. Therefore, all rankings should be considered exploratory (as stated above). Second, there is a significant disparity in the number of studies included for each outcome, with only four studies (335 participants) included for fatigue and five for anxiety, but a larger number of studies for sleep-related outcomes. Effect estimates for outcomes with smaller sample sizes are less stable, resulting in a “low” certainty in the GRADE rating. Third, all included studies reported only short-term follow-up outcomes (mostly ≤ 12 weeks). The long-term efficacy of these non-pharmacological interventions among nightshift nurses remains unclear, and it is impossible to assess the impact of these interventions on long-term workplace outcomes such as occupational burnout and turnover intention. Fourth, most original studies did not report data on intervention adherence, fidelity, or adverse events. The real-world effectiveness may be lower than in trial settings. Fifth, subgroup analyses or meta-regressions on different “dose” parameters (e.g., light intensity, essential oil concentration, number of treatment sessions, and total duration) within the same intervention category were not conducted, and the optimal parameters for each intervention protocol remain to be determined. Sixth, since most interventions were only reported in a single study, the issue of heterogeneity within categories had a limited impact in this analysis. However, the level of detail in the descriptions of intervention protocols varied across the original studies. It is recommended that future studies adopt standardized reporting using the Template for Intervention Description and Replication (TIDieR) checklist to facilitate more refined cross-study comparisons. Seventh, the coverage of gray literature in this study remains insufficient, and some studies with negative results may have been omitted. Furthermore, due to the absence of a closed loop in the network structure and the limited number of studies at each node, it was not possible to conduct a formal statistical assessment of publication bias using methods such as comparison-adjusted funnel plots. Additionally, the original studies included in this NMA generally lacked standardized assessment and reporting of fidelity to the intervention and participant adherence. The RoB2 assessment indicated that most studies had a low risk of bias in the deviations from intended interventions domain, which indirectly suggested no serious deviations in intervention implementation. Nevertheless, it was impossible to determine whether each intervention was strictly implemented according to the planned protocol or to quantify the actual level of engagement of participants with the intervention. The absence of fidelity and adherence assessments may affect the interpretation of the results of this study in two ways: insufficient fidelity may lead to an underestimation of the effectiveness of the interventions, and variations in adherence across studies may represent a potential source of heterogeneity. Therefore, the effect sizes observed in this study should be interpreted with caution, and future studies should adopt standardized tools to assess intervention fidelity and report detailed adherence data.

Based on the findings and limitations of this study, future research should prioritize the following areas. First, head-to-head RCTs should be conducted to directly compare the most promising interventions (e.g., SIP vs. APT, REBT vs. MTI, BLE vs. PPEO) to verify whether the exploratory ranking generated in this study can be confirmed in direct comparisons. Besides, interventions that demonstrated favorable effects in East Asian studies (particularly APT and specific aromatherapy) should be systematically replicated and validated within Western healthcare settings to assess whether cultural context serves as an effect modifier. Moreover, trials should be designed with long-term follow-up (≥6 months) to assess the long-term benefits and safety of interventions. Low-cost, low-intensity digital intervention protocols (e.g., app-delivered simplified CBT-i modules, self-guided mindfulness audio) should also be explored to address scheduling compatibility issues for nightshift nurses and improve real-world accessibility and sustainability. Regarding the clinical significance of effect sizes, we interpreted the SMDs for each intervention according to Cohen's d criteria (large effect: BLE, REBT, SIP, SSHI, MTI; moderate effect: APT, PPEO) and used the minimum clinically important difference (MCID) values of commonly used scales, such as the PSQI (MCID approximately 1.5–3.0 points) and Insomnia Severity Index (MCID approximately 6–8 points), as supplementary criteria for assessment. Meanwhile, we specifically note that the very large effect sizes observed for BLE and REBT each stem from a single study with a limited sample size, raising the possibility of overestimation; therefore, these findings should be interpreted with caution. Regarding the sustainability of effects, we conducted a stratified discussion based on the mechanisms of action of each intervention. Interventions based on cognitive-behavioral change (dCBT, REBT, MTI) may yield more lasting effects because they alter cognitive patterns and behavioral habits. Evidence from the literature also supports that the efficacy of CBT-i can be maintained for 6–12 months. Physical environmental interventions (BLE, WLD) rely on continued application, and their effects may diminish after discontinuation. Currently, there is a lack of high-quality evidence from long-term follow-up studies for aromatherapy and APT. We recommend that future studies include a follow-up period of at least 6 months to verify whether short-term benefits can translate into sustained clinical improvement.

## 5 Conclusion

This NMA provides a comprehensive exploration of comparative evidence to date on non-pharmacological interventions for sleep and occupational stress among nightshift nurses. SIP, MTI, APT, REBT, and PPEO showed the highest exploratory rankings in total sleep scores, sleep duration, depression, anxiety, and fatigue, respectively. However, all findings are based on indirect evidence from a star-shaped network and should be regarded as exploratory and hypothesis-generating rather than confirmatory conclusions. The core value of this study lies in that it identifies the most promising intervention strategies to guide future head-to-head RCTs, rather than providing definitive recommendations for current clinical practice.

## Data Availability

The original contributions presented in the study are included in the article/Supplementary material, further inquiries can be directed to the corresponding author.
