# Supplementary material for: Non-pharmacological therapies for sleep and occupational stress regulation among nightshift nurses: a systematic review and Bayesian network meta-analysis based on randomized controlled trials
Source: Front Public Health. 2026 Jul 24;14:1882224. doi: 10.3389/fpubh.2026.1882224 (PMC13447436; doi:10.3389/fpubh.2026.1882224)
Supplement: Supplementary file 1 [file Data_Sheet_1.docx]

**Supplementary Table 1.** Search strategy.

| **Pubmed** | | |
| --- | --- | --- |
| # | Query | Results |
| 1 | "Nurses"[Mesh] | 104362 |
| 2 | anaesthesist nurse assistant[Title/Abstract] OR anesthetist nurse assistant[Title/Abstract] OR community health nurses[Title/Abstract] OR nurse, community health[Title/Abstract] OR Nurse, Registered[Title/Abstract] OR nurses[Title/Abstract] OR nurses, community health[Title/Abstract] OR nurses, public health[Title/Abstract] OR Nurses, Registered[Title/Abstract] OR nursing assistance[Title/Abstract] OR Nursing Personnel[Title/Abstract] OR Personnel, Nursing[Title/Abstract] OR public health nurses[Title/Abstract] OR Registered Nurses[Title/Abstract] | 253158 |
| 3 | #1 OR #2 | 316616 |
| 4 | night[Title/Abstract] OR shift[Title/Abstract] | 415995 |
| 5 | #3 AND #4 | 7100 |
| 6 | sleep[Title/Abstract] OR stress[Title/Abstract] OR anxiety[Title/Abstract] OR depress[Title/Abstract] OR fatigue[Title/Abstract] | 1763459 |
| 7 | #5 AND #6 | 1983 |
| 8 | randomized controlled trial[Title/Abstract] OR controlled clinical trial[Title/Abstract] OR placebo[Title/Abstract] OR random allocation[Title/Abstract] OR randomized[Title/Abstract] OR Cohort Study[Title/Abstract] OR Clinical Trial[Title/Abstract] | 1420617 |
| 9 | #7 AND #8 | 154 |

| **Embase** | | |
| --- | --- | --- |
| # | Query | Results |
| 1 | 'nurse'/exp | 252666 |
| 2 | 'anaesthesist nurse assistant':ab,ti OR 'anesthetist nurse assistant':ab,ti OR 'community health nurses':ab,ti OR 'nurse, community health':ab,ti OR 'nurse, registered':ab,ti OR nurses:ab,ti OR 'nurses, community health':ab,ti OR 'nurses, public health':ab,ti OR 'nurses, registered':ab,ti OR 'nursing assistance':ab,ti OR 'nursing personnel':ab,ti OR 'personnel, nursing':ab,ti OR 'public health nurses':ab,ti OR 'registered nurses':ab,ti | 309286 |
| 3 | #1 OR #2 | 438854 |
| 4 | night:ab,ti OR shift:ab,ti | 518438 |
| 5 | #3 AND #4 | 11056 |
| 6 | sleep:ab,ti OR stress:ab,ti OR anxiety:ab,ti OR depress:ab,ti OR fatigue:ab,ti | 2318718 |
| 7 | #5 AND #6 | 3091 |
| 8 | 'randomized controlled trial':ab,ti OR 'controlled clinical trial':ab,ti OR placebo:ab,ti OR 'random allocation':ab,ti OR randomized:ab,ti OR 'cohort study':ab,ti OR 'clinical trial':ab,ti | 2278271 |
| 9 | #7 AND #8 | 370 |

| **Cochrane Library** | | |
| --- | --- | --- |
| # | Query | Results |
| 1 | MeSH descriptor: [Nursing] explode all trees | 4426 |
| 2 | (anaesthesist nurse assistant OR anesthetist nurse assistant OR community health nurses OR nurse, community health OR Nurse, Registered OR nurses OR nurses, community health OR nurses, public health OR Nurses, Registered OR nursing assistance OR Nursing Personnel OR Personnel, Nursing OR public health nurses OR Registered Nurses):ti,ab,kw | 24204 |
| 3 | #1 OR #2 | 26946 |
| 4 | (night OR shift):ti,ab,kw | 32360 |
| 5 | #3 AND #4 | 859 |
| 6 | (sleep OR stress OR anxiety OR depress OR fatigue):ti,ab,kw | 243415 |
| 7 | #5 AND #6 | 445 |
| 8 | (randomized controlled trial OR controlled clinical trial OR placebo OR random allocation OR randomized OR Cohort Study OR Clinical Trial):ti,ab,kw | 1484978 |
| 9 | #7 AND #8 | 304 |

| **Web of science** | | |
| --- | --- | --- |
| # | Query | Results |
| 4 | anaesthesist nurse assistant OR anesthetist nurse assistant OR community health nurses OR nurse, community health OR Nurse, Registered OR nurses OR nurses, community health OR nurses, public health OR Nurses, Registered OR nursing assistance OR Nursing Personnel OR Personnel, Nursing OR public health nurses OR Registered Nurses (主题) | 427567 |
| 5 | night OR shift (主题) | 1628687 |
| 6 | sleep OR stress OR anxiety OR depress OR fatigue (主题) | 3979380 |
| 7 | randomized controlled trial OR controlled clinical trial OR placebo OR random allocation OR randomized OR Cohort Study OR Clinical Trial (主题) | 2824553 |
| 8 | #7 AND #6 AND #5 AND #4 | 547 |

| **CNKI** | |
| --- | --- |
| Query | Results |
| 夜班护士AND焦虑 + 抑郁 +压力 +疲劳 + 睡眠 | 227 |

**Supplementary Table 2** League table - total sleep score

| RC | **-1.4 (-1.89, -0.91)** | **-1.5 (-1.87, -1.13)** | -0.5 (-1.09, 0.09) | **-3.2 (-3.83, -2.57)** | **-0.6 (-1.05, -0.15)** | -0.5 (-1.03, 0.03) | **-0.5 (-0.91, -0.09)** | **-0.5 (-0.91, -0.09)** | -0.3 (-0.87, 0.27) | **-1 (-1.53, -0.47)** | **-1.6 (-2.03, -1.17)** |
| --- | --- | --- | --- | --- | --- | --- | --- | --- | --- | --- | --- |
| **1.4 (0.91, 1.89)** | REOI | -0.1 (-0.71, 0.52) | 0.9 (0.13, 1.66) | **-1.8 (-2.59, -1.01)** | 0.8 (0.13, 1.46) | 0.9 (0.18, 1.61) | 0.9 (0.26, 1.54) | 0.9 (0.26, 1.54) | 1.1 (0.35, 1.85) | 0.4 (-0.32, 1.13) | -0.2 (-0.85, 0.45) |
| **1.5 (1.13, 1.87)** | 0.1 (-0.52, 0.71) | SSHI | 1 (0.3, 1.7) | **-1.7 (-2.43, -0.97)** | 0.9 (0.31, 1.48) | 1 (0.36, 1.64) | 1 (0.45, 1.55) | 1 (0.45, 1.56) | 1.2 (0.52, 1.88) | 0.5 (-0.14, 1.15) | -0.1 (-0.67, 0.47) |
| 0.5 (-0.09, 1.09) | -0.9 (-1.66, -0.13) | -1 (-1.7, -0.3) | DCBT | **-2.7 (-3.56, -1.84)** | -0.1 (-0.84, 0.64) | 0 (-0.79, 0.8) | 0 (-0.71, 0.72) | 0 (-0.71, 0.72) | 0.2 (-0.62, 1.01) | -0.5 (-1.29, 0.29) | **-1.1 (-1.82, -0.37)** |
| **3.2 (2.57, 3.83)** | **1.8 (1.01, 2.59)** | **1.7 (0.97, 2.43)** | **2.7 (1.84, 3.56)** | BLE | 2.6 (1.83, 3.37) | 2.7 (1.88, 3.52) | 2.7 (1.96, 3.45) | 2.7 (1.95, 3.45) | 2.9 (2.06, 3.75) | 2.2 (1.38, 3.01) | 1.6 (0.84, 2.36) |
| **0.6 (0.15, 1.05)** | -0.8 (-1.46, -0.13) | -0.9 (-1.48, -0.31) | 0.1 (-0.64, 0.84) | -2.6 (-3.37, -1.83) | WLD | 0.1 (-0.59, 0.8) | 0.1 (-0.51, 0.71) | 0.1 (-0.51, 0.72) | 0.3 (-0.42, 1.03) | -0.4 (-1.09, 0.3) | **-1 (-1.62, -0.38)** |
| 0.5 (-0.03, 1.03) | -0.9 (-1.61, -0.18) | -1 (-1.64, -0.36) | 0 (-0.8, 0.79) | -2.7 (-3.52, -1.88) | -0.1 (-0.8, 0.59) | MHP | 0 (-0.67, 0.67) | 0 (-0.67, 0.67) | 0.2 (-0.57, 0.98) | -0.5 (-1.25, 0.25) | **-1.1 (-1.78, -0.42)** |
| **0.5 (0.09, 0.91)** | -0.9 (-1.54, -0.26) | -1 (-1.55, -0.45) | 0 (-0.72, 0.71) | -2.7 (-3.45, -1.96) | -0.1 (-0.71, 0.51) | 0 (-0.67, 0.67) | APT | 0 (-0.58, 0.58) | 0.2 (-0.49, 0.9) | -0.5 (-1.17, 0.17) | **-1.1 (-1.69, -0.51)** |
| **0.5 (0.09, 0.91)** | -0.9 (-1.54, -0.26) | -1 (-1.56, -0.45) | 0 (-0.72, 0.71) | -2.7 (-3.45, -1.95) | -0.1 (-0.72, 0.51) | 0 (-0.67, 0.67) | 0 (-0.58, 0.58) | MTI | 0.2 (-0.5, 0.9) | -0.5 (-1.17, 0.17) | **-1.1 (-1.69, -0.51)** |
| 0.3 (-0.27, 0.87) | -1.1 (-1.85, -0.35) | -1.2 (-1.88, -0.52) | -0.2 (-1.01, 0.62) | -2.9 (-3.75, -2.06) | -0.3 (-1.03, 0.42) | -0.2 (-0.98, 0.57) | -0.2 (-0.9, 0.49) | -0.2 (-0.9, 0.5) | AM | -0.7 (-1.47, 0.08) | **-1.3 (-2.01, -0.59)** |
| **1 (0.47, 1.53)** | -0.4 (-1.13, 0.32) | -0.5 (-1.15, 0.14) | 0.5 (-0.29, 1.29) | -2.2 (-3.01, -1.38) | 0.4 (-0.3, 1.09) | 0.5 (-0.25, 1.25) | 0.5 (-0.17, 1.17) | 0.5 (-0.17, 1.17) | 0.7 (-0.08, 1.47) | LI-LED | -0.6 (-1.28, 0.08) |
| **1.6 (1.17, 2.03)** | 0.2 (-0.45, 0.85) | 0.1 (-0.47, 0.67) | **1.1 (0.37, 1.82)** | -1.6 (-2.36, -0.84) | **1 (0.38, 1.62)** | **1.1 (0.42, 1.78)** | **1.1 (0.51, 1.69)** | **1.1 (0.51, 1.69)** | **1.3 (0.59, 2.01)** | 0.6 (-0.08, 1.28) | SIP |

Table note: RC: Routine Care; REOI: Rosemary Essential Oil Inhalation; SSHI: Structured Sleep Hygiene Intervention; DCBT: Digital Cognitive Behavioral Therapy; BLE: Bright Light Exposure; WLD: Wearable Light Device; MHP: Mobile Health Program; APT: Auricular; Plaster Therapy; MTI: Mindfulness Training Intervention; AM: Aromatherapy Massage; LI-LED: Low-Intensity Light-Emitting Diode; SIP: Sleep Intervention Program.

**Supplementary Table 3** League table - sleep duration

| RC | 0 (-0.53, 0.53) | -0.2 (-0.73, 0.33) | **-0.71 (-1.01, -0.4)** | **-1 (-1.43, -0.57)** | -0.1 (-0.65, 0.45) | **-0.6 (-0.97, -0.23)** |
| --- | --- | --- | --- | --- | --- | --- |
| 0 (-0.53, 0.53) | BLE | -0.2 (-0.96, 0.55) | **-0.71 (-1.32, -0.1)** | **-1 (-1.68, -0.32)** | -0.1 (-0.86, 0.67) | -0.6 (-1.24, 0.05) |
| 0.2 (-0.33, 0.73) | 0.2 (-0.55, 0.96) | MHP | -0.51 (-1.12, 0.1) | **-0.8 (-1.48, -0.12)** | 0.1 (-0.67, 0.86) | -0.4 (-1.05, 0.24) |
| **0.71 (0.4, 1.01)** | **0.71 (0.1, 1.32)** | 0.51 (-0.1, 1.12) | APT | -0.29 (-0.82, 0.23) | 0.61 (-0.02, 1.24) | 0.11 (-0.37, 0.58) |
| **1 (0.57, 1.43)** | **1 (0.32, 1.68)** | **0.8 (0.12, 1.48)** | 0.29 (-0.23, 0.82) | MTI | 0.9 (0.2, 1.6) | 0.4 (-0.17, 0.97) |
| 0.1 (-0.45, 0.65) | 0.1 (-0.67, 0.86) | -0.1 (-0.86, 0.67) | -0.61 (-1.24, 0.02) | -0.9 (-1.6, -0.2) | AM | -0.5 (-1.17, 0.16) |
| **0.6 (0.23, 0.97)** | 0.6 (-0.05, 1.24) | 0.4 (-0.24, 1.05) | -0.11 (-0.58, 0.37) | -0.4 (-0.97, 0.17) | 0.5 (-0.16, 1.17) | SIP |

Table note: RC: Routine Care; BLE: Bright Light Exposure; MHP: Mobile Health Program; APT: Auricular Plaster Therapy; MTI: Mindfulness Training Intervention; AM: Aromatherapy Massage; SIP: Sleep Intervention Program.

**Supplementary Table 4** League table - depression

| RC | -0.5 (-1.08, 0.09) | -0.3 (-1.04, 0.44) | **-0.6 (-1.03, -0.16)** | 0 (-0.31, 0.31) | -0.4 (-0.89, 0.09) | 0.34 (-0.15, 0.83) |
| --- | --- | --- | --- | --- | --- | --- |
| 0.5 (-0.09, 1.08) | DCBT | 0.2 (-0.25, 0.65) | -0.1 (-0.83, 0.63) | 0.5 (-0.16, 1.17) | 0.1 (-0.66, 0.87) | 0.84 (0.08, 1.6) |
| 0.3 (-0.44, 1.04) | -0.2 (-0.65, 0.25) | SHE | -0.3 (-1.16, 0.56) | 0.3 (-0.5, 1.1) | -0.1 (-0.98, 0.79) | 0.64 (-0.24, 1.52) |
| **0.6 (0.16, 1.03)** | 0.1 (-0.63, 0.83) | 0.3 (-0.56, 1.16) | APT | 0.6 (0.06, 1.13) | 0.2 (-0.46, 0.85) | 0.94 (0.28, 1.6) |
| 0 (-0.31, 0.31) | -0.5 (-1.17, 0.16) | -0.3 (-1.1, 0.5) | -0.6 (-1.13, -0.06) | ISWM | -0.4 (-0.98, 0.18) | 0.34 (-0.24, 0.93) |
| 0.4 (-0.09, 0.89) | -0.1 (-0.87, 0.66) | 0.1 (-0.79, 0.98) | -0.2 (-0.85, 0.46) | 0.4 (-0.18, 0.98) | LI-LED | 0.74 (0.05, 1.44) |
| -0.34 (-0.83, 0.15) | -0.84 (-1.6, -0.08) | -0.64 (-1.52, 0.24) | -0.94 (-1.6, -0.28) | -0.34 (-0.93, 0.24) | -0.74 (-1.44, -0.05) | RSSCI |

Table note: RC: Routine Care; DCBT: Digital Cognitive Behavioral Therapy; SHE: Sleep Hygiene Education; APT: Auricular Plaster Therapy; ISWM: Individualized Shift Work Management; LI-LED: Low-Intensity Light-Emitting Diode; RSSCI: Reflective and Social Support-based Confiding Intervention

**Supplementary Table 5** League table - anxiety

| RC | -0.5 (-1.09, 0.09) | **-1.5 (-1.97, -1.03)** | **-0.5 (-0.93, -0.07)** | -0.1 (-0.41, 0.21) | **-3 (-3.59, -2.42)** |
| --- | --- | --- | --- | --- | --- |
| 0.5 (-0.09, 1.09) | DCBT | **-1 (-1.75, -0.25)** | 0 (-0.73, 0.73) | 0.4 (-0.26, 1.07) | **-2.5 (-3.34, -1.67)** |
| **1.5 (1.03, 1.97)** | **1 (0.25, 1.75)** | BLE | 1 (0.36, 1.64) | 1.4 (0.83, 1.97) | **-1.5 (-2.25, -0.75)** |
| **0.5 (0.07, 0.93)** | 0 (-0.73, 0.73) | -1 (-1.64, -0.36) | APT | 0.4 (-0.13, 0.94) | **-2.5 (-3.22, -1.77)** |
| 0.1 (-0.21, 0.41) | -0.4 (-1.07, 0.26) | -1.4 (-1.97, -0.83) | -0.4 (-0.94, 0.13) | ISWM | **-2.9 (-3.57, -2.23)** |
| **3 (2.42, 3.59)** | **2.5 (1.67, 3.34)** | **1.5 (0.75, 2.25)** | **2.5 (1.77, 3.22)** | **2.9 (2.23, 3.57)** | REBT |

Table note: RC: Routine Care; DCBT: Digital Cognitive Behavioral Therapy; BLE: Bright Light Exposure; APT: Auricular Plaster Therapy; ISWM: Individualized Shift Work Management; REBT: Rational Emotive Behavior Therapy

**Supplementary Table 6** League table - fatigue

| RC | -0.3 (-0.75, 0.15) | -0.2 (-0.73, 0.33) | -0.06 (-0.44, 0.31) | **-0.7 (-1.15, -0.25)** |
| --- | --- | --- | --- | --- |
| 0.3 (-0.15, 0.75) | WLD | 0.1 (-0.6, 0.79) | 0.24 (-0.35, 0.82) | -0.4 (-1.04, 0.23) |
| 0.2 (-0.33, 0.73) | -0.1 (-0.79, 0.6) | MHP | 0.14 (-0.51, 0.79) | -0.5 (-1.2, 0.19) |
| 0.06 (-0.31, 0.44) | -0.24 (-0.82, 0.35) | -0.14 (-0.79, 0.51) | ELE-MLA | **-0.64 (-1.22, -0.06)** |
| **0.7 (0.25, 1.15)** | 0.4 (-0.23, 1.04) | 0.5 (-0.19, 1.2) | **0.64 (0.06, 1.22)** | PPEO |

Table note: RC: Routine Care; WLD: Wearable Light Device; MHP: Mobile Health Program; ELE-MLA: Evening Light Exposure and Morning Light Avoidance; PPEO: Pure Pelargonium Essential Oil.

**Supplementary Table** **7** GRADE Evidence Quality Assessment Table

| **Certainty assessment** | | | | | | | **Certainty** |
| --- | --- | --- | --- | --- | --- | --- | --- |
| **№ of studies** | **Study design** | **Risk of bias** | **Inconsistency** | **Indirectness** | **Imprecision** | **Other considerations** |  |
| **Sleep Scores** | | | | | | | |
| 14 | randomised trials | not serious | serious^a^ | not serious | not serious | none | ⨁⨁⨁◯ Moderate^a^ |
| **Sleep Duration** | | | | | | | |
| 12 | randomised trials | not serious | serious^a^ | not serious | not serious | none | ⨁⨁⨁◯ Moderate^a^ |
| **Depression** | | | | | | | |
| 12 | randomised trials | not serious | serious^a^ | not serious | not serious | none | ⨁⨁⨁◯ Moderate^a^ |
|  | | | | | | | |
| **Anxiety** \| \| \| \| \| \| \|; 5 \| randomised trials \| not serious \| serious^a^ \| not serious \| serious^b^ \| none \| ⨁⨁◯◯ Low^ab^ | | | | | | | |
| **Fatigue** \| \| \| \| \| \| \|; 4 \| randomised trials \| not serious \| serious^a^ \| not serious \| serious^b^ \| none \| ⨁⨁◯◯ Low^ab^ | | | | | | | |

**CI:** confidence interval

Explanations

a. The network structure does not contain any closed loops, and the inconsistency check has not been carried out.
b. The number of included studies was limited (<10), and the 95% confidence interval for the effect estimate was relatively wide, suggesting some degree of imprecision.

**Supplementary Table 8** Sensitivity Analysis

Supplementary Table 8-1 Ranking Table of SUCRA Before and After Elimination of Low-Quality Studies on Sleep Scores

| Not excluded | | excluded | |
| --- | --- | --- | --- |
| treament | SUCRA | treament | SUCRA |
| RC | 0.023 | RC | 0.027 |
| REOI | 0.77 | **REOI** | **0.801** |
| **SSHI** | **0.811** | **SSHI** | **0.839** |
| DCBT | 0.331 | DCBT | 0.351 |
| **BLE** | **0.999** | **BLE** | **0.999** |
| WLD | 0.399 | WLD | 0.418 |
| MHP | 0.332 | MHP | 0.349 |
| MTI | 0.332 | MTI | 0.351 |
| AM | 0.209 | AM | 0.223 |
| LI-LED | 0.612 | LI-LED | 0.641 |
| **SIP** | **0.847** |  |  |
| APT | 0.332 |  |  |

Supplementary Table 8-2 Ranking Table of SUCRA Before and After Excluding Low-Quality Studies on Sleep Duration

| Not excluded | | excluded | |
| --- | --- | --- | --- |
| treament | SUCRA | treament | SUCRA |
| RC | 0.182 | RC | 0.182 |
| BLE | 0.207 | BLE | 0.206 |
| MHP | 0.375 | MHP | 0.366 |
| **APT** | **0.787** | **APT** | **0.951** |
| **MTI** | **0.959** | **MTI** | **0.860** |
| AM | 0.291 | AM | 0.286 |
| **SIP** | **0.699** | **SIP** | **0.648** |
